# Predicting bleeding risk in PAD patients on antiplatelets using TEG coagulation testing

**DOI:** 10.26502/jsr.10020456

**Published:** 2025-07-22

**Authors:** Adriana A. Rodriguez Alvarez, Isabella Ferlini Cieri, Mounika Boya, Shiv Patel, Anahita Dua

**Affiliations:** Division of Vascular and Endovascular Surgery, Massachusetts General Hospital, Boston, MA 02114 USA.

**Keywords:** Hemorrhage, Peripheral arterial disease, Thromboelastography, Antiplatelet therapy, Bleeding risk, Platelet function testing, Revascularization

## Abstract

**Introduction:**

Bleeding is a major concern while using antithrombotic therapy. While Thromboelastography with Platelet Mapping (TEG-PM) predicts postoperative bleeding and platelet dysfunction in trauma, its utility in peripheral artery disease (PAD) remains unclear. Hence, this study aimed to evaluate whether platelet inhibition (PI) and maximum amplitude of adenosine diphosphate (MA ADP) can predict bleeding risk in PAD.

**Methods:**

Patients with PAD undergoing lower extremity revascularization between 2021–2025 were prospectively evaluated and monitored for one year to identify bleeding events. Bleeding events were defined as clinically significant hemorrhages that required medical intervention or transfusion. Patients were stratified based on the occurrence of bleeding, and descriptive statistics characterized each group. The Mann-Whitney U test assessed differences in platelet function, while receiver operating characteristic (ROC) analysis determined the optimal TEG-PM cutoff values for predicting increased bleeding risk.

**Results:**

A total of 234 patients were analyzed, of whom 14 (5%) experienced a bleeding event. The bleeding cohort exhibited higher platelet inhibition (94.5% vs. 24.1%; p<0.0001) and lower MA ADP (22.4 vs. 52; p<0.0001), suggesting reduced platelet aggregation and clot strength. ROC analysis revealed platelet inhibition >86.4% (AUC: 0.89, sensitivity: 71%, specificity: 92%) and MA ADP <31.9 (AUC: 0.85, sensitivity: 79%, specificity: 85%) as predictive thresholds for bleeding risk.

**Conclusion:**

High platelet inhibition (>86.4%) and low MA ADP (<31.9%) may serve as indicators of bleeding risk in PAD patients on antiplatelets, highlighting the potential utility of TEG-PM in guiding personalized antithrombotic management.

## Introduction

Peripheral artery disease (PAD) affects over 200 million people globally, with prevalence rising significantly with age. While often asymptomatic, PAD is associated with a high risk of amputation and elevated mortality, with a five-year death rate exceeding 80 per 1,000 patient-years. 1,2 To reduce vascular events, current guidelines recommend long-term single antiplatelet therapy (SAPT) with low-dose aspirin or clopidogrel, or dual antiplatelet therapy (DAPT) combining both agents after endovascular revascularizations.^3^,^4^ However, bleeding complications are common, especially with DAPT, making optimal management unclear and largely based on expert opinion.^5^,^6^ The CHARISMA trial showed that while dual therapy reduced ischemic events, it also raised minor bleeding risks compared to aspirin alone, suggesting a potential benefit in high-risk ischemic PAD patients with low bleeding risk.^7^ Similarly, the PEGASUS-TIMI 54 trial showed that ticagrelor plus aspirin lowered major cardiovascular events but substantially increased major bleeding risks, 2–3 times higher than aspirin alone.^7^ These findings highlight the complex challenge of balancing therapeutic efficacy with bleeding risks in PAD management.

The emergence of thromboelastography with platelet mapping (TEG-PM) provides a more comprehensive assessment of hemostasis by quantitatively and graphically evaluating clot formation dynamics, strength, and stability.⁷ Building on this capability, our research group previously established an optimal thrombosis cutoff using ROC analysis to evaluate platelet aggregation, platelet inhibition (PI), and maximum amplitude of adenosine diphosphate (MA ADP) in patients with PAD undergoing revascularization and on antiplatelet therapy. Platelet aggregation exhibited an area under the curve AUC of 0.769 (95% CI, 0.684–0.853) at a 70.8% cutoff, with 87% sensitivity and 70% specificity. Platelet inhibition showed an AUC of 0.756 (95% CI, 0.670–0.841) at a 29.2% cutoff, achieving 87% sensitivity and 71% specificity.^8^ Increased clot strength was predictive of thrombosis or stenosis within 30 days, with an MA ADP cutoff >42 mm.^9^ Having established reliable thresholds for thrombotic risk, our current focus is to identify an optimal bleeding cutoff using similar Receiver operating characteristic ROC analysis. This is particularly important given the clinical challenge of balancing thrombosis prevention with bleeding risk in patients on antiplatelet therapy—especially those with PAD, who often require long-term or intensified antiplatelet regimens. Therefore, we hypothesize that ROC curve analysis will identify specific cutoff values for PI and MA ADP that effectively differentiate between patients at high risk of bleeding in PAD. The aim of this study is to determine the optimal cutoff values of PI and MA ADP for predicting bleeding risk in patients with PAD) using ROC curve analysis.

## Material and methods

### Study design and ethics

We conducted a prospective single-institution study that included 234 patients undergoing lower extremity revascularization for PAD from December 2021 through February 2025 at a single large tertiary institution. The study was conducted in accordance with the ethical principles outlined in the Declaration of Helsinki and received approval from the Mass General Brigham Institutional Review Board (IRB) prior to initiation. Written informed consent was obtained from all patients prior to any data collection.

Patients who were undergoing revascularization were approached for discussion of enrollment in the research study. Patients above the age of 18, diagnosed with PAD, and who underwent successful lower extremity revascularization were included in the study. Participants who were unable to undergo serial blood draws or had contraindications or allergies to antiplatelet therapy were excluded from the study.

Successful revascularization was considered when adequate blood flow was restored to an ischemic area by bypassing and treating the atherosclerotic blockage using methods such as angioplasty, stenting, or bypass surgery.

### Patient data

Baseline demographic data was collected from the electronic medical record (EMR), including age at the time of intervention, race, sex at birth, body mass index (BMI), and history of major comorbidities such as hypertension, hyperlipidemia, coronary artery disease (CAD), myocardial infarction (MI), diabetes, chronic kidney disease (CKD), pulmonary embolism (PE), stroke, and deep venous thromboembolism (DVT).

### Patient stratification

Patients were stratified into two groups based on the occurrence of a bleeding event. A bleeding event was defined as clinically significant hmorrhages requiring medical intervention, transfusion of blood products, or unplanned hospitalization, along with the discontinuation of antiplatelet therapy.

### Sample collection and processing

Blood samples for TEG-PM analysis were collected in 4.0 mL non-gel sodium heparin Vacutainers at four time points: baseline (before revascularization) and at one, three-, and six-months post-procedure. For the purposes of this research, the TEG results chosen were as follows: for the non-bleeding group, the result from the last follow-up visit was used; for the bleeding group, the TEG result closest to the date of the bleeding event was selected.

Following manufacturer guidelines, all samples underwent a mandatory 30-minute incubation period and were analyzed within two hours of collection.

### Thromboelastography with Platelet Mapping Assay (TEG-PM)

Coagulation profiles were generated using the TEG^®^ 6s Hemostasis Analyzer (Haemonetics Corporation, Boston, MA, USA), where the TEG-Platelet Mapping 6s System with PlateletMapping^®^ cartridges measured kinetic changes during clotting of heparinized whole blood samples to provide qualitative platelet function assessment. The PlateletMapping^®^ cartridge evaluated maximal amplitude (MA) of blood clots under four distinct conditions: kaolin activation (MAHKH), which stimulated maximal thrombin response to measure complete clot strength; fibrin-only measurement (MAActF), which used reptilase to directly convert fibrinogen to fibrin while inhibiting thrombin; adenosine diphosphate challenge (MAADP) and arachidonic acid challenge (MAAA), both of which measured MA when platelets were stimulated by their respective agonists while thrombin was inhibited. Reduced MA responses to ADP or AA indicated P2Y12 inhibitor or aspirin action, respectively, with results calculated as a percentage of platelet aggregation or inhibition for both ADP and AA pathways.

### Statistical analysis

Descriptive statistics were calculated for all variables. The Shapiro-Wilk test was used to assess the normality of continuous data. Continuous variables were reported as means with standard deviations and categorical variables were summarized as frequencies and percentages. Group comparisons for continuous variables were performed using the Mann-Whitney U test due to non-normal distribution, while categorical variables were compared using Fisher’s exact test.

Time to event was defined as the duration from the date of the procedure to the last follow-up visit for participants in the non-bleeding group, and from the date of the procedure to the date of the bleeding event for those in the bleeding group.

(ROC) curve analysis was performed to determine the optimal cutoff values of TEG-PM parameters for predicting increased bleeding risk. The (AUC) with 95% confidence intervals was calculated to assess discriminatory performance.

A p-value of <0.05 was considered statistically significant. All statistical analyses were performed in R Studio Version 2023.09.0+463.^10^

### Interventions and medication data

Details regarding the type of revascularization intervention were collected and categorized as open surgical procedures, endovascular interventions, or combined approaches. Open procedures included bypass grafting and endarterectomy, while endovascular interventions comprised angioplasty with or without stenting. Combined approaches involved hybrid procedures utilizing both open and endovascular techniques during the same operative session.

Antithromboprophylaxis therapy was documented for all patients and classified into six categories: mono antiplatelet therapy (MAPT, typically aspirin or clopidogrel alone), dual antiplatelet therapy (DAPT, usually aspirin plus clopidogrel or another P2Y12 inhibitor), direct oral anticoagulant (DOAC) monotherapy, MAPT plus DOAC, DAPT plus DOAC, or no antithrombotic therapy. Medication regimens were recorded at baseline and at each follow-up visit, with any changes in therapy documented. For patients who experienced bleeding events, the antithrombotic regimen in use at the time of bleeding was recorded for analysis.

All interventions and medication changes were performed according to standard clinical practice guidelines and institutional protocols, with dosing adjustments made based on patient-specific factors including age, weight, and renal function.

## Results

### Demographics

A total of 229 patients were analyzed, of whom 16 (7.0%) experienced a bleeding event. The median age was slightly higher in the bleeding group compared to the non-bleeding group (69.00 [13.00] vs. 68.00 [13.25]; p = 0.82). Sex distribution was similar between groups, with males representing the majority in both cohorts (68.8% in bleeding vs. 67.1% in non-bleeding; p = 0.299). The majority of patients were White (87.5% in bleeding vs. 83.56% in non-bleeding), followed by smaller proportions of Black, Asian, Hispanic, and other/unknown races (p = 1.0). Body mass index (BMI) was lower in the bleeding group (median 26.51 [6.92] vs. 29.27 [7.07]; p = 0.59).

Although comorbidities such as diabetes (43.8% vs. 62.44%), hypertension (100% vs. 89.67%), and hyperlipidemia (81.2% vs. 88.73%) were less prevalent in the bleeding group, these differences were not statistically significant. Notably, coronary artery disease was more common in the bleeding group (75% vs. 61.03%; p = 0.26). Renal function, tobacco use, and history of myocardial infarction (37.5% vs. 30.66%; p = 0.23), deep vein thrombosis (12.5% vs. 15.02%; p = 1.0), or stroke (6.2% vs. 18.30%; p = 1.0) did not differ significantly between groups.

Regarding intervention type, there were similar distributions of open (25.00% vs. 24.88%), endovascular (6.25% vs. 59.62%), and combined procedures (12.50% vs. 15.49%) between the bleeding and non-bleeding groups (p = 0.73). The distribution of antithromboprophylaxis therapy also showed no significant differences between groups (p = 0.51), with DAPT being the most common in the bleeding group (43.8%).

Time to event was significantly shorter in the bleeding group (1.15 months vs. 4.89 months; p < 0.0001) (Table 1).

### Platelet function assessed by TEG-PM

**Figure 1:**
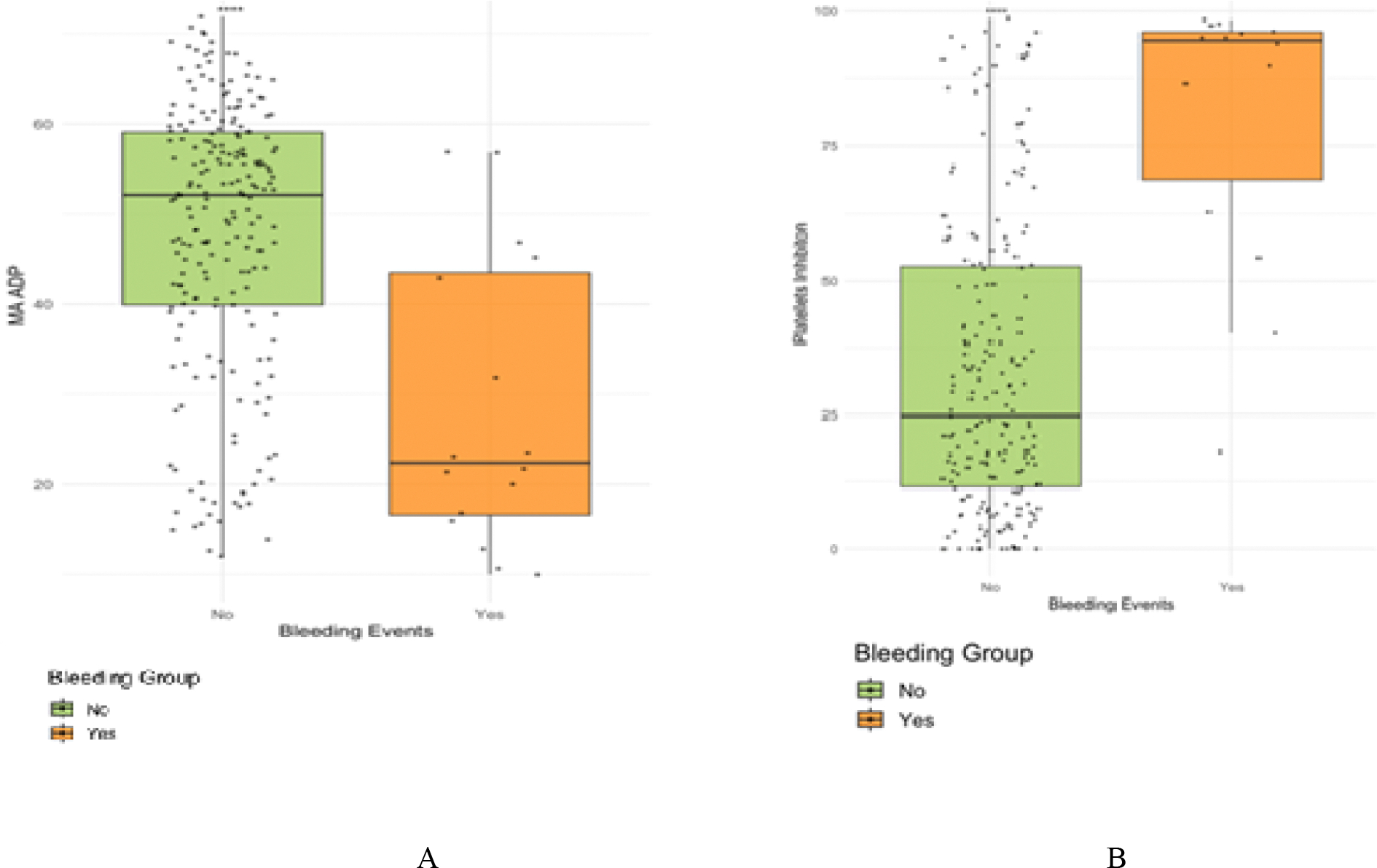
Comparison of platelet function between non-bleeding group and bleeding group. Comparison of clot strength, measured by adenosine diphosphate maximum amplitude (ADP MA), between bleeding and non-bleeding groups. **(B)** Comparison of platelet inhibition levels between bleeding and non-bleeding groups.

The bleeding group had a lower median ADP MA than the non-bleeding group (22.35mm vs. 52.10 mm, p<0.0001), and higher platelet inhibition (94.50% vs. 24.50%, <0.0001) (Figure 1).

### Receiver Operating Characteristic (ROC) curve

**Figure 2:**
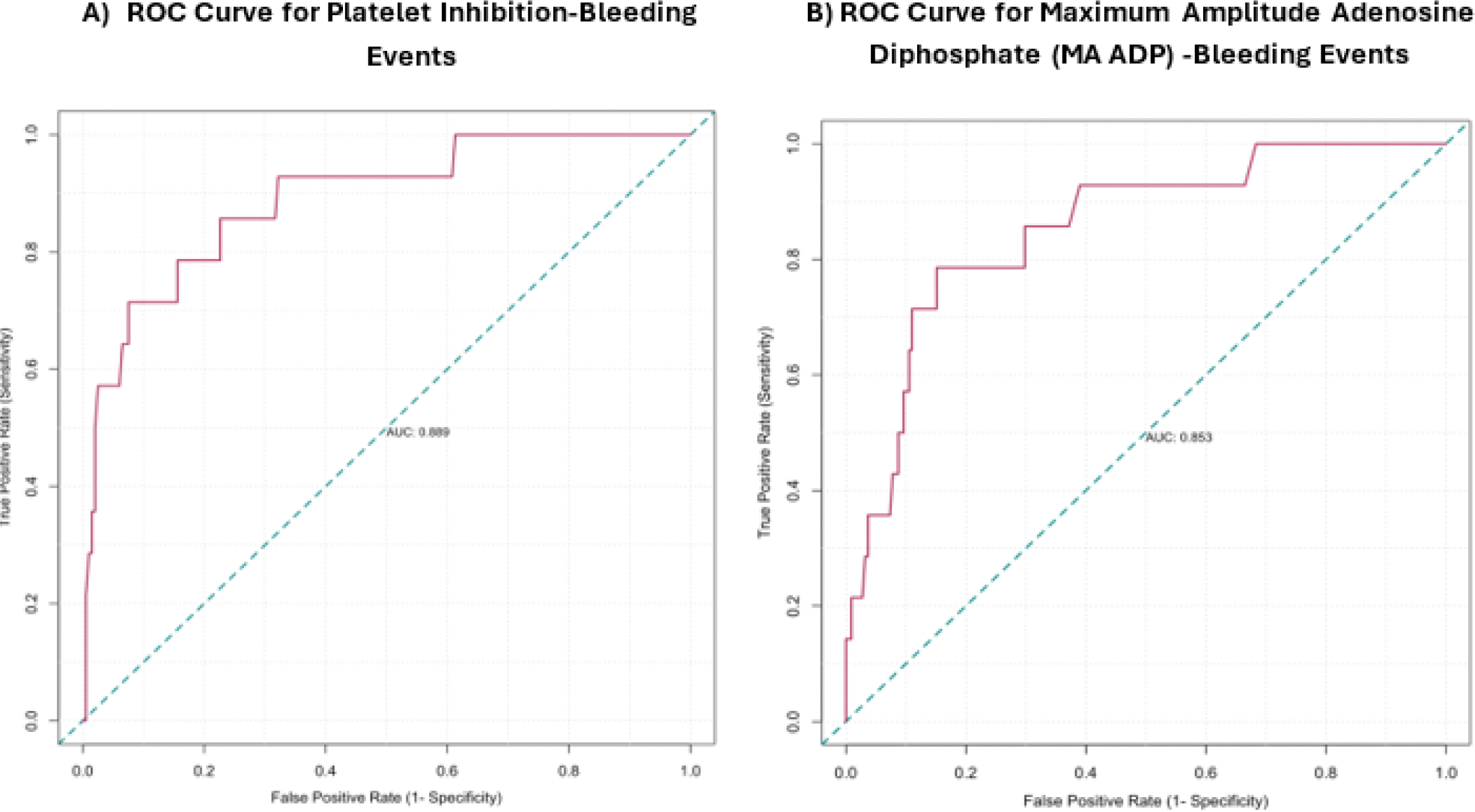
Receiver operating characteristic (ROC) analysis determined the optimal TEG-PM cutoff values for predicting increased bleeding risk **(A) ROC curve analysis for platelet inhibition** bleeding risk [AUC: 0.89, cutoff: 86.4, sensitivity: 0.71, specificity: 0. 92, 95% confidence of interval [CI]: 0.79–0.98,] **(B) ROC curve analysis for maximum amplitude adenosine diphosphate (MA ADP)** bleeding risk [AUC: 0.85, cutoff: 31.9, sensitivity: 0.79, specificity: 0. 85, 95% CI: 0.69– 0.92]

ROC analysis revealed platelet inhibition >86.4% (AUC: 0.89, sensitivity: 71%, specificity: 92%, 95% confidence interval [CI]: 0.79--0.98) and MA ADP <31.9 (AUC: 0.85, sensitivity: 79%, specificity: 85%, 95% CI: 0.69–0.92) as predictive thresholds for bleeding risk (Figure 2).

### Kaplan Meier Curve

**Figure 3:**
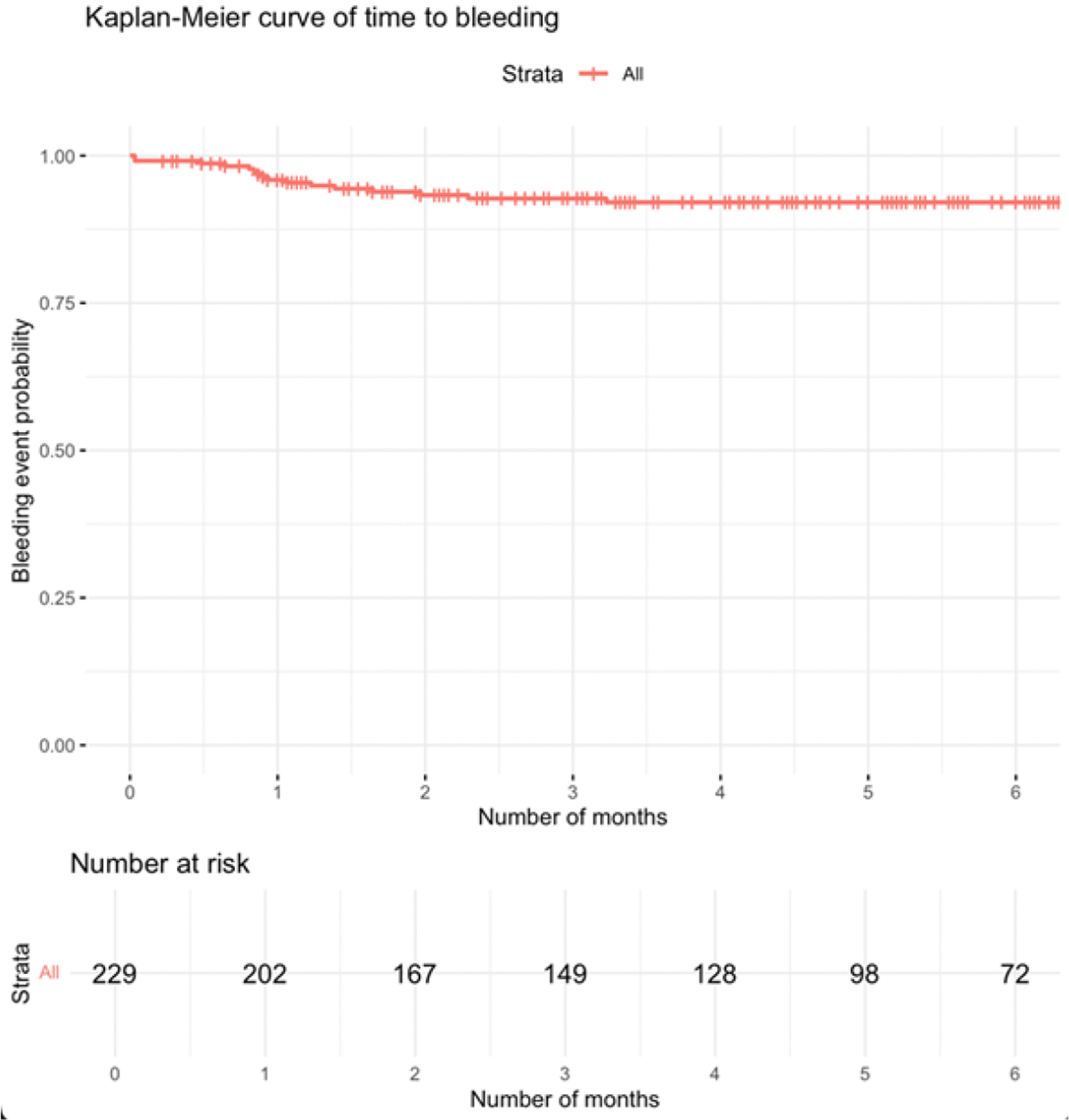
Kaplan-Meier curve showing time to first bleeding event over a 6-month follow-up period. The red line represents the cumulative probability of remaining free from bleeding among all patients (n = 229). Vertical tick marks indicate censored observations. The number of patients at risk is displayed below the x-axis at monthly intervals.

The Kaplan-Meier analysis of 229 patients showed a high probability of remaining free from bleeding events over 6 months (Figure 3). Most bleeding occurred within the first 2--3 months, after which the curve plateaued.

## Discussion

In this cohort of 229 patients, 6.8% experienced a bleeding event. Patients in the bleeding group were slightly older and had significantly higher BMI and a greater prevalence of coronary artery disease. Although other comorbidities, such as diabetes, hypertension, and renal dysfunction, were more common in the non-bleeding group, these differences were not statistically significant. Notably, the time to event was significantly shorter in those who bled. Platelet function assessed by TEG-PM revealed significantly lower ADP MA and higher platelet inhibition in the bleeding group, supporting a strong association between platelet dysfunction and bleeding risk. ROC analysis identified platelet inhibition >86.4% and ADP MA <31.9 mm as predictive thresholds, with high sensitivity and specificity, highlighting the potential utility of TEG-PM in bleeding risk stratification.

This study demonstrates that TEG-PM parameters such as platelet inhibition >86.4% and ADP MA <31.9, can effectively predict bleeding risk in patients with PAD undergoing lower extremity revascularization. These findings represent a significant advancement in the personalized management of antithrombotic therapy for PAD patients. The observed association between high platelet inhibition and low ADP MA values in patients who experienced bleeding events aligns with previous research in other vascular territories, particularly in coronary artery disease [11,12]. However, our study is among the first to establish specific cutoff values for these parameters in the PAD population. The high sensitivity and specificity of these thresholds suggest that TEG-PM could serve as a valuable clinical tool for bleeding risk stratification.

This research addresses a critical gap in PAD management. Balancing the efficacy of antithrombotic therapy against bleeding risk remains challenging, particularly in high-bleeding-risk patients. The CHARISMA, PLATO, and PEGASUS-TIMI 54 trials have demonstrated the benefits of various antiplatelet regimens in reducing major adverse cardiovascular events, but all showed increased bleeding risk with more aggressive antithrombotic approaches.^13^–^15^ Our findings suggest that TEG-PM could help clinicians navigate this complex risk-benefit assessment on an individualized basis.

TEG-PM is a promising tool for bleeding risk assessment in PAD patients, potentially enabling clinicians to tailor antithrombotic therapy based on individual platelet function profiles. Implementation of TEG-PM in clinical practice could represent a significant step toward precision medicine in PAD management, optimizing the balance between thrombotic and bleeding risk.

Several limitations should be acknowledged. First, despite the prospective design and year-long follow-up, the relatively low incidence of bleeding events limits the power of our analysis. Second, we did not account for variations in antiplatelet regimens or dosing, which could influence both TEG-PM parameters and bleeding outcomes. Additionally, the small sample size in the bleeding group, along with an imbalanced distribution of sex and race, may limit the generalizability of the findings. Future research should validate these cutoff values in larger, multicenter cohorts and explore whether TEG-PM-guided therapy modification can reduce bleeding events without compromising antithrombotic efficacy.

## Conclusions

Bleeding events were associated with older age, higher BMI, and a greater prevalence of coronary artery disease. TEG-PM revealed significantly impaired platelet function in this group, with high platelet inhibition and low ADP MA emerging as strong predictors of bleeding risk. These results highlight the potential role of TEG-PM in guiding antiplatelet therapy by identifying patients who may be over-inhibited and therefore at increased risk of bleeding. Integrating platelet function testing into PAD management could allow for more individualized antiplatelet strategies, balancing the need for thrombosis prevention with the risk of bleeding.

## Figures and Tables

**Table 1:** Effect of time with different events in different groups.

Variables	Non-bleeding events	Bleeding	p-value
	(n = 213)	(n =16)	
**Age, median [IQR]**	68.00 [13.25]	69.00 [13.00]	*0.82* ^a^
**Sex, n (%)**			*0.29* ^b^
Women	70 (32.9)	5 (31.2)	
Men	143 (67.1)	11 (68.8)	
**Race, n (%)**			*1* ^b^
White	178 (83.56)	14 (87.5)	
Black	17 (7.99)	0 (0)	
Asian	8 (3.76)	0 (0)	
Hispanic	9 (4.22)	0 (0)	
Other/unknown	1 (0.47)	2 (12.4)	
**BMI, median [IQR]**	29.27 [7.07]	26.51 [6.92]	*0.59* ^a^
**Diabetes, n (%)**	133 (62.44)	7 (43.8)	*0.59* ^a^
**Hypertension, n (%)**	191 (89.67)	16 (100)	*0.48* ^a^
**Renal status**			*0.89* ^a^
GFR >90	69 (32.39)	8 (50)	
GFR 60 to 89	72 (33.80)	1 (6.2)	
GFR 30 to 59	45 (21.12)	7 (43.8)	
GFR 15–29	4 (1.87)	0 (0)	
GFR <15 (on dialysis)	23 (10.79)	0 (0)	
**Hyperlipidemia, n (%)**	189 (88.73)	13 (81.2)	*1:00 AM*
**Coronary Artery Disease, n (%)**	130 (61.03)	12 (75)	*0.26* ^a^
**History of Myocardial Infarction, n (%)**	65 (30.66)	6 (37.5)	*0.23* ^a^
**History of DVT, n (%)**	32 (15.02)	2 (12.5)	*1:00 AM*
**History of Stroke, n (%)**	39 (18.30)	1 (6.2)	*1:00 AM*
**Tobacco Use, n (%)**			*0.13* ^a^
Never	54 (25.35)	1 (6.2)	
Former	114 (53.52)	9 (56.2)	
Current	45 (21.12)	6 (37.5)	
**Type of intervention, n (%)**			*0.73* ^a^
Open	53 (24.88)	4 (25.00)	
Endovascular	127 (59.62)	10 (6.25)	
Combined	33 (15.49)	2 (12.50)	
**Anti thromboprophylaxis therapy, n (%)**			*0.51* ^a^
MAPT	26 (12.06)	1 (6.2)	
DAPT	60 (28.16)	7 (43.8)	
DOAC	5 (2.34)	0 (0)	
MAPT+DOAC	66 (30.98)	2 (12.5)	
DAPT+ DOAC	54 (25.35)	6 (37.5)	
None	2 (1)	0 (0)	
**Time to event; months**	4.89	1.15	<0.0001* ^a^

BMI: body mass index; GFR: glomerular filtration rate; DVT: deep vein thrombosis; MAPT = Mono antiplatelet therapy (aspirin/clopidogrel); DAPT = dual antiplatelet therapy (Aspirin + Clopidogrel, Aspirin + Ticagrelor); DOAC = direct oral anticoagulants (Apixaban, Rivaroxaban); MAPT + DOAC = Mono antiplatelet therapy and direct oral anticoagulants (Aspirin + Apixaban, Aspirin + Rivaroxaban, Clopidogrel + Rivaroxaban); DAPT + DOAC = dual antiplatelet therapy and direct oral anticoagulants (Aspirin + Clopidogrel + Ticagrelor, Aspirin + Clopidogrel + Rivaroxaban, Aspirin + Clopidogrel + Apixaban). Time to event was defined as the duration from the date of the procedure to the last follow-up visit for participants in the non-bleeding group, and from the date of the procedure to the date of the bleeding event for those in the bleeding group.

* A p-value of <0.05 was considered statistically significant.

a Fisher’s exact test was used for categorical variables.

b Mann-Whitney U test was used for continuous variables.
